# Efficient base editing with expanded targeting scope using an engineered Spy-mac Cas9 variant

**DOI:** 10.1038/s41421-019-0128-4

**Published:** 2019-12-03

**Authors:** Zhiquan Liu, Huanhuan Shan, Siyu Chen, Mao Chen, Yuning Song, Liangxue Lai, Zhanjun Li

**Affiliations:** 1 Key Laboratory of Zoonosis Research, Ministry of Education, College of Animal Science, Jilin University, 130062 Changchun, China; 2 CAS Key Laboratory of Regenerative Biology, Guangdong Provincial Key Laboratory of Stem Cell and Regenerative Medicine, South China Institute for Stem Cell Biology and Regenerative Medicine, Guangzhou Institutes of Biomedicine and Health, Chinese Academy of Sciences, 510530 Guangzhou, China; 3 Guangzhou Regenerative Medicine and Health Guang Dong Laboratory (GRMH-GDL), 510005 Guangzhou, China; 4 Institute for Stem Cell and Regeneration, Chinese Academy of Sciences, 100101 Beijing, China

**Keywords:** Genomic analysis, DNA recombination

Dear Editor,

The clustered regularly interspaced short palindromic repeat (CRISPR) system, including newly developed base editing technology, has exhibited powerful genome manipulation capability^1^. Base editors that can achieve targeted C-to-T (CBE) or A-to-G (ABE) conversions without generating DNA double-strand breaks (DSBs) or requiring a donor template represent significant advances in both disease modeling and gene therapy^2,3^. However, the conventional *Streptococcus pyogenes* Cas9 (Spy Cas9) requires a protospacer adjacent motif (PAM) of NGG, which limits the applicability of base editors that are highly dependent on the PAMs suitably adjacent to target bases. In addition, although some natural and engineered Cas9 variants with different PAM specificities have been utilized in base editors, such as representative Cpf1 (TTTV (V = A/G/C))^4^ and SpCas9-NG (NG)^5^, their targeting scope is still limited to genomic regions rich in G or T bases. Recently, Spy-mac Cas9 was generated by rationally exchanging the PAM-interacting (PI) region of the conventional Spy Cas9 with that of the newly discovered *Streptococcus macacae* Cas9 (Smac Cas9), showing 5′-NAA-3′ PAM specificity and possessing efficient gene editing in human cells^6^. In this study, we demonstrated the effectiveness of the Spy-mac Cas9-assisted cytidine and adenine base editors Spy-mac BE4max and Spy-mac ABEmax, and found that 5′-TAAA-3′ is the only high-efficiency PAM for Spy-mac Cas9 observed in this study.

To obtain the best efficiency of base editing, the Spy-mac Cas9 system was combined with the current optimal version of the base editors BE4max and ABEmax^7^ to generate Spy-mac BE4max and Spy-mac ABEmax, respectively (Fig. 1a). To fully evaluate the PAM specificity and editing efficiency, we first tested the Spy-mac BE4max system in rabbit embryos at 16 target sites, including all NAAN PAMs, as a proof of concept (Supplementary Table S1). Base editing was conducted in rabbit pronuclear-stage embryos by microinjecting of Spy-mac BE4max-encoding mRNA and single-guide RNA (sgRNA). Base editing frequencies were evaluated by Sanger sequencing and T-A cloning. Notably, the average C-to-T editing frequency at the *Tyr-1* site with TAAA PAM was high at 86.00 ± 8.72%, while much lower efficiencies ranging from 13.33 ± 6.67% to 26.67 ± 11.74% were observed at the other five sites with AAAT, GAAG, CAAG, AAAC, and CAAC PAMs (Fig. 1b and Supplementary Fig. S1). However, no obvious base editing events were observed at most tested sites (10/16), consistent with variations in the targeting efficiencies of Spy-mac Cas9 at different targeting sites in human cells^6^ (Fig. 1b and Supplementary Fig. S1).

**Fig. 1:**
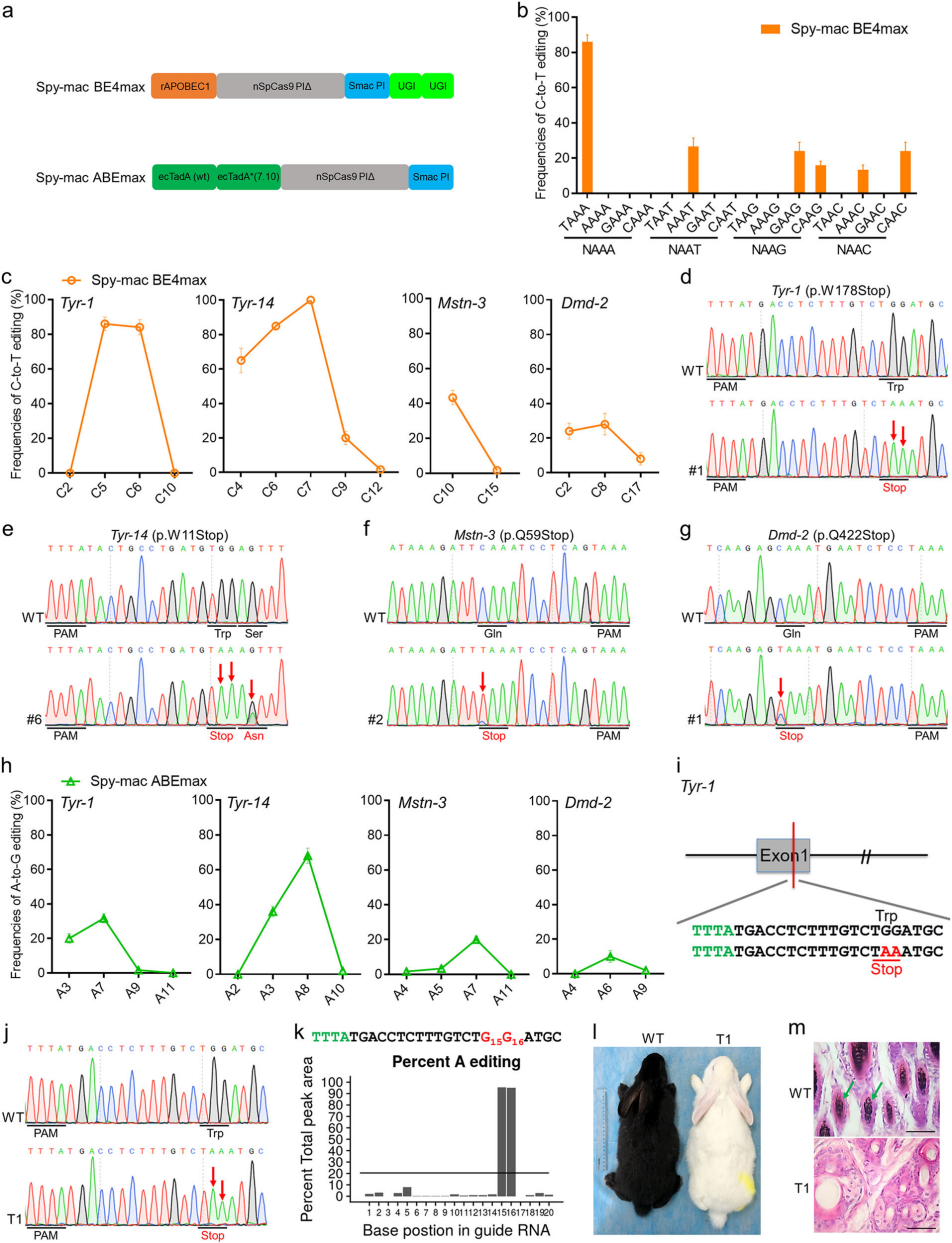
Spy-mac BE4max and Spy-mac ABEmax induce efficient C-to-T/A-to-G base editing in vivo. **a** Schematic representation of Spy-mac BE4max and Spy-mac ABEmax architecture. PIΔ, deletion of PI domain. **b** Average frequencies of C-to-T base editing at seven target sites that included NAAA and TAAN PAMs by Spy-mac BE4max in rabbit blastocysts. Data are presented as mean ± SEM. (*n* = ~6 blastocysts). **c** Frequencies of single C-to-T conversions at four target sites with TAAA PAMs by Spy-mac BE4max in rabbit blastocysts. Data are presented as mean ± SEM. (*n* = ~6 blastocysts). **d**–**g** Representative sequencing chromatograms of edited rabbit blastocysts at four target sites using the Spy-mac BE4max system. Targeted C·G to T·A conversions (red arrows). The relevant codon identities at the target site are presented under the DNA sequence. **h** Frequencies of single A-to-G conversions at four target sites with TAAA PAM by Spy-mac ABEmax in rabbit blastocysts. Data are presented as mean ± SEM. (*n* = ~6 blastocysts). **i** The target sequence at the *Tyr-1* locus using the Spy-mac BE4max system. The PAM and sgRNA target sequences are shown in green and black, respectively. Target mutation (red). **j** Representative sequencing chromatograms from a WT and mutant rabbit (T1). The red arrow indicates the substituted nucleotide. The relevant codon identities at the target site are presented under the DNA sequence. **k** The predicted editing bar plot based on Sanger sequencing chromatograms from T1 by EditR. **l** Photograph of WT and *Tyr-1* mutant (T1) rabbits at 1 month. **m** H&E staining of skin from WT and T1 rabbits. The green arrows highlight the melanin in the basal layer of the epidermis of WT rabbits. Scale bars: 50 μm

Encouraged by the results of the pilot study, we examined whether the Spy-mac Cas9 variant may primarily target TAAA PAM in rabbit embryos. Therefore, another three sites (*Tyr-5*, *Mstn-3*, and *Dmd-2*) with TAAA PAMs were designed to verify our hypothesis (Supplementary Table S1). Remarkably, all sites showed efficient C-to-T conversions, with average editing frequencies ranging from 28.00 ± 13.93% at *Dmd-2* to 100.00 ± 0.00% at *Tyr-14* (Fig. 1c and Supplementary Fig. S2). Moreover, targeted C·G to T·A conversions were successfully achieved to induce stop codons at all four sites, as expected (Fig. 1d, g). In particular, the homozygous p.W178Stop- or p.W11Stop-targeted mutations were determined at *Tyr-1* or *Tyr-14* (Fig. 1d, e). In addition, only a few unwanted by-products, including indels and non-C-to-T conversions, were observed in mutant embryos at *Tyr-1* (1/10, #3) and *Dmd-2* (1/10, #1) using Spy-mac BE4max (Supplementary Figs. S1, 2), which was consistent with the high product purity of the BE4max architecture in human cells^7^. Despite the high efficiency of Spy-mac BE4max-mediated C-to-T conversion, additional base editing tool such as recently reported ABE system was also tested here with TAAA PAMs at the four sites. Notably, site-specific A·T to G·C conversions were observed at all tested sites, with average editing frequencies ranging from 10.00 ± 7.75% at *Dmd-2* to 68.00 ± 9.70% at *Tyr-14* (Fig. 1h and Supplementary Figs. S3, 4). Taken together, these results demonstrated that both Spy-mac BE4max and Spy-mac ABEmax could induce efficient C-to-T/A-to-G base editing in rabbit embryos with TAAA PAMs.

Oculocutaneous albinism type 1 (OCA1) in humans results from mutations in the *Tyr* gene, which encodes tyrosinase, the key enzyme in pigment biosynthesis in mammals^8^. Patients with OCA1 have been reported to have impaired tyrosinase activity and a consequential absence of pigmentation^9^. Here, two target G-to-A conversions were designed in exon 1 of the rabbit *Tyr* gene to yield a premature stop codon (p.W178stop) (Fig. 1i). After the microinjection of Spy-mac BE4max-encoding mRNA and sgRNA^10^, the rabbit embryos were transplanted into the surrogate mother, and subsequently five pups were obtained (Supplementary Table S2). Strikingly, T-A cloning showed that four of these pups (80%) were homozygous with nonsense mutations (p.W178stop) at the target site (Fig. 1j, k and Supplementary Fig. S5). In addition, no indels or non-C-to-T mutations were detected in the Founder (F0) rabbits, which demonstrated the high product purity of Spy-mac BE4max (Supplementary Fig. S5). As expected, the mutants exhibited a complete albino phenotype, consistent with their mutant genotype (Fig. 1l). Furthermore, histological haematoxylin-eosin (H&E) staining revealed the absence of melanin in the hair follicles of the T1 mutant, but not in their wild-type (WT) littermates (Fig. 1m). In addition, no obvious off-target mutations were detected at potential off-target sites in mutant rabbits by using Sanger sequencing and T7E1 cleavage assays^11,12^ (Supplementary Fig. S6). Overall, these results demonstrated that Spy-mac BE4max successfully mediated the *Tyr-1* p.W178stop mutation in F0 rabbit with high efficiency, which precisely recapitulates the pathological features of human OCA1.

In summary, we first demonstrated that the Spy-mac Cas9-assisted cytidine and adenine base editors Spy-mac BE4max and Spy-mac ABEmax can induce efficient C-to-T/A-to-G conversions in vivo. In addition, the observed PAM scope of Spy-mac Cas9 is not 5′-NAA-3′ as previously reported in human cells but in reality only 5′-TAAA-3′ manifests as high-efficiency PAM in this study. Moreover, Spy-mac BE4max can induce targeted base editing in F0 rabbits with high efficiency to precisely mimic human pathology condition. Thus, Spy-mac Cas9-assisted base editors with expanded targeting scopes are promising tools for establishing animal models and developing precise gene therapy in the future.

## Supplementary information

Supplementary Information

## Supplementary information

**Supplementary Information** accompanies the paper at (10.1038/s41421-019-0128-4).
