# Supplementary material for: Efficient base editing with expanded targeting scope using an engineered Spy-mac Cas9 variant
Source: Cell Discov. 2019 Dec 3;5:58. doi: 10.1038/s41421-019-0128-4 (PMC6888851; doi:10.1038/s41421-019-0128-4)
Supplement: Supplementary file 1 — Supplementary Information [file 41421_2019_128_MOESM1_ESM.docx]

**Materials and methods**

**Ethics statement**

New Zealand white rabbits and Lianshan black rabbits were obtained from the Laboratory Animal Center of Jilin University (Changchun, China). All animal studies were conducted according to the experimental practices and standards approved by the Animal Welfare and Research Ethics Committee at Jilin University.

**Plasmid construction**

BE4max and ABEmax were obtained from Addgene (#112093 and #112095). The PI domain of Smac Cas9 was synthesized and cloned into BE4max or ABEmax by Genscript Biotech (Nanjing) to obtain Spy-mac BE4max or Spy-mac ABEmax, respectively. The amino acid sequences of the plasmids are listed in the Supplementary Sequences.

**mRNA and gRNA preparation**

All plasmids were linearized with NotI and transcribed *in vitro* using the HiScribe™ T7 ARCA mRNA kit (NEB). The mRNA was purified using the RNeasy Mini Kit (Qiagen) according to the manufacturer’s protocol. The sgRNA oligos were annealed into pUC57-sgRNA expression vectors containing a T7 promoter. The sgRNAs were then amplified and transcribed *in vitro* using the MAXIscript T7 Kit (Ambion) and purified using the miRNeasy Mini Kit (Qiagen) according to the manufacturer’s protocol. The sgRNA oligo sequences used in this study are listed in Table S3.

**Microinjection of rabbit zygotes**

The protocol used for the microinjection of pronuclear-stage embryos has been described in detail in our previously published study^10^. Briefly, a mixture of mRNA (200 ng/µl) and sgRNA (50 ng/µl) was co-injected into the cytoplasm of pronuclear zygotes. The injected embryos were transferred to EBSS medium for short-term culture at 38.5 °C, 5% carbon dioxide and 100% humidity. Then, approximately 30–50 injected zygotes were transferred into the oviducts of recipient rabbits.

**Single-embryo PCR amplification and rabbit genotyping**

Each group was injected with an average of approximately 10 embryos to test the base editing efficiency. The injected embryos were transferred to EBSS medium for culture at 38.5 °C, 5% carbon dioxide and 100% humidity. Then, the injected embryos were collected at the blastocyst stage. Genomic DNA was extracted in embryo lysis buffer (1% NP40) at 56 °C for 60 minutes and then at 95 °C for 10 minutes in a BIO-RAD PCR Thermocycler. The extracted products were amplified by PCR (pre-degeneration at 95°C for 5 min , 42 cycles at 95°C for 30 s, 58°C for 30 s, and 72°C for 30 s, with a final extension at 72°C, for 5 min) and determined by Sanger sequencing and T-A cloning. The genomic DNA of newborn rabbits was extracted from ear clips and analysed by PCR genotyping, Sanger sequencing and T-A cloning. All primers used for genotyping are listed in Table S4.

**Off-target assay**

The top ten potential off-target sites (POTs) in the rabbit genome for sgRNA were predicted to analyse site-specific edits according to Cas-OFFinder^11^ (http://www.rgenome.net/cas-offinder/). The PCR products of the POTs were sequenced and confirmed by T7E1 enzyme digestion, as previously described^12^. All primers for the off-target assay are listed in Table S5.

**Haematoxylin and eosin (H&E) staining**

The dorsal skin from WT and mutant rabbits was fixed in 4% paraformaldehyde for 48 hours, embedded in paraffin wax and then sectioned for slides. The slides were stained with H&E and viewed under a Nikon ts100 microscope.

**Statistical analysis**

All data are expressed as the mean ± SEM, with at least three individual determinations in all experiments. The data were analysed using GraphPad prism software 6.0.


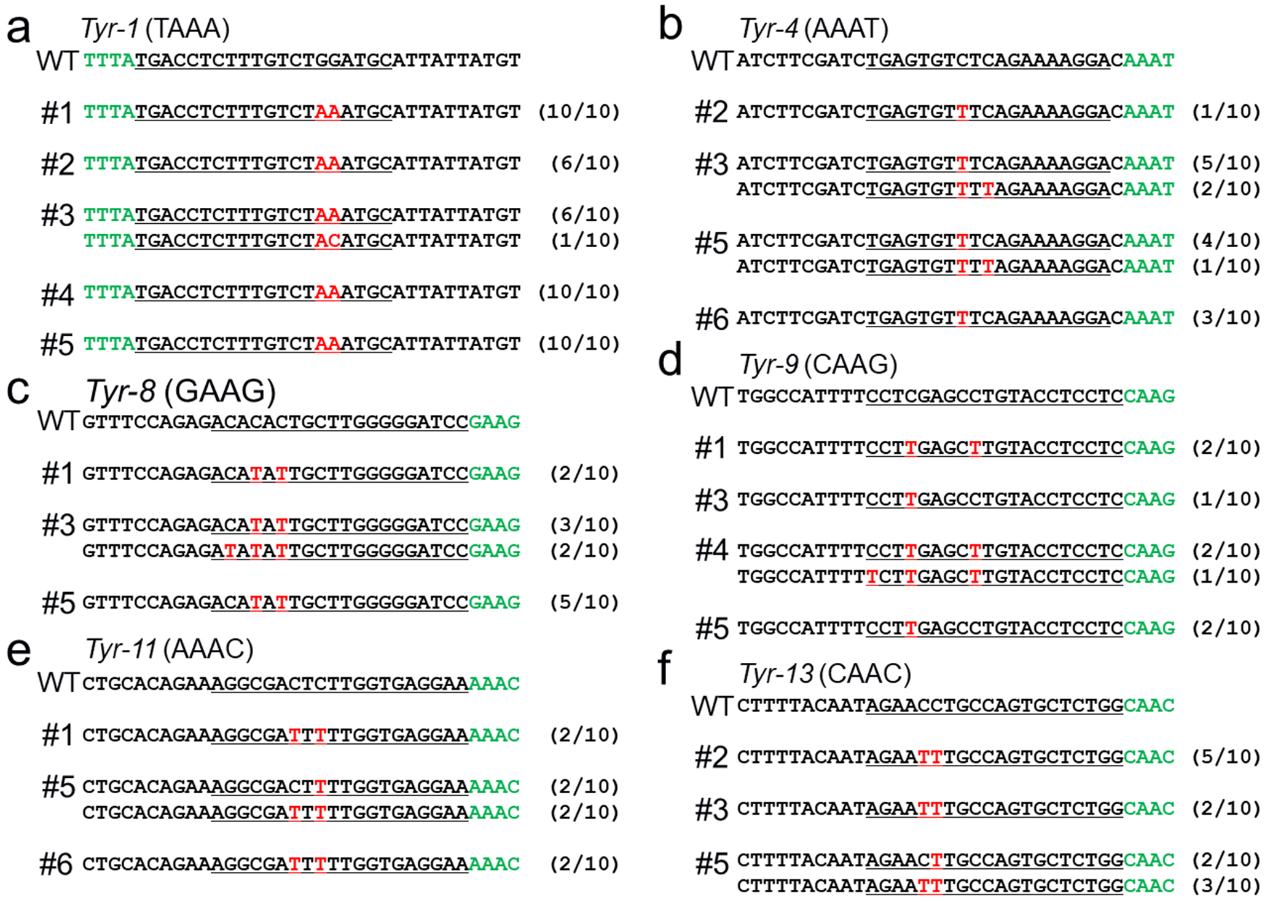


**Figure S1.** Base editing frequencies and product distribution at sixteen target sites that included all NAAN PAMs in rabbit blastocysts using Spy-mac BE4max. The number of clones for each sequence pattern is indicated. The target sequence (underlined), PAM region (green) and substituted nucleotides (red) are shown. WT: wild type. #1-#6: each mutant blastocyst used for T-A cloning. N/N indicates positive colonies out of the total sequenced.


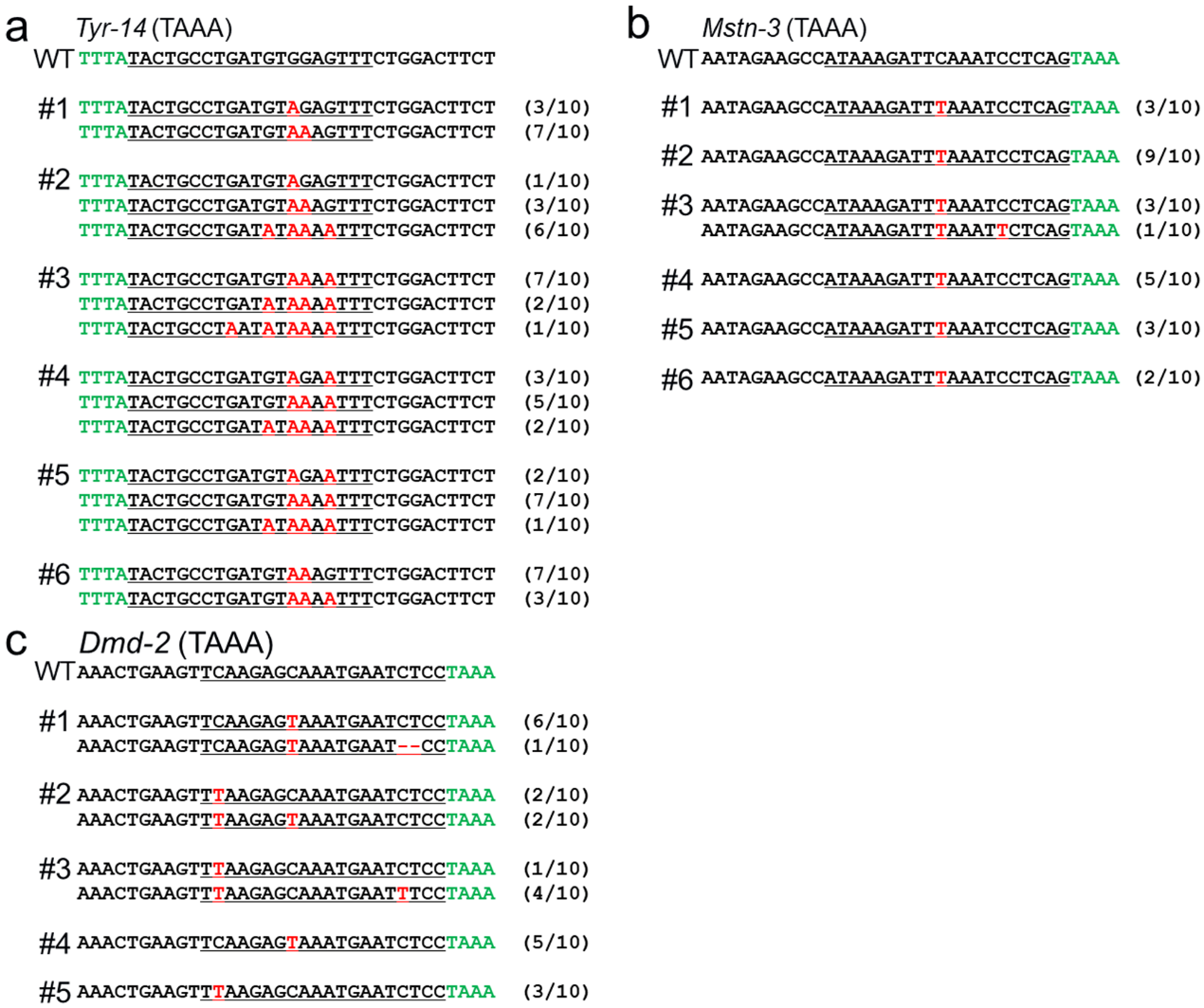


**Figure S2.** Base editing frequencies and product distribution at *Tyr-14*, *Mstn-3* and *Dmd-2* in rabbit blastocysts using Spy-mac BE4max. The number of clones for each sequence pattern is indicated. The target sequence (underlined), PAM region (green) and substituted nucleotides (red) are shown. WT: wild type. #1-#6: each mutant blastocyst used for T-A cloning. N/N indicates positive colonies out of the total sequenced.


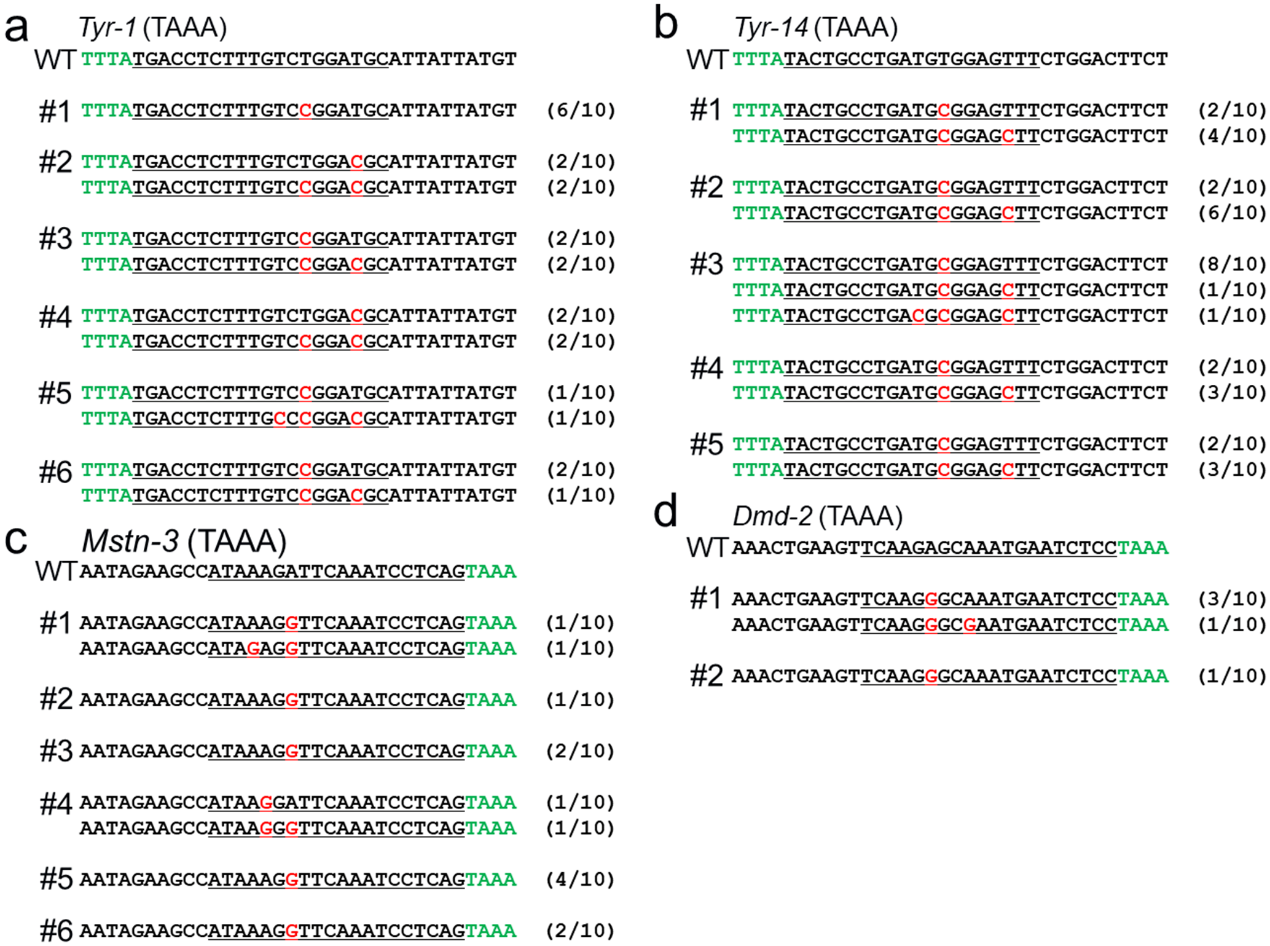


**Figure S3.** Base editing frequencies and product distribution at *Tyr-1*, *Tyr-14*, *Mstn-3* and *Dmd-2* in rabbit blastocysts using Spy-mac ABEmax. The number of clones for each sequence pattern is indicated. The target sequence (underlined), PAM region (green) and substituted nucleotides (red) are shown. WT: wild type. #1-#6: each mutant blastocyst used for T-A cloning. N/N indicates positive colonies out of the total sequenced.


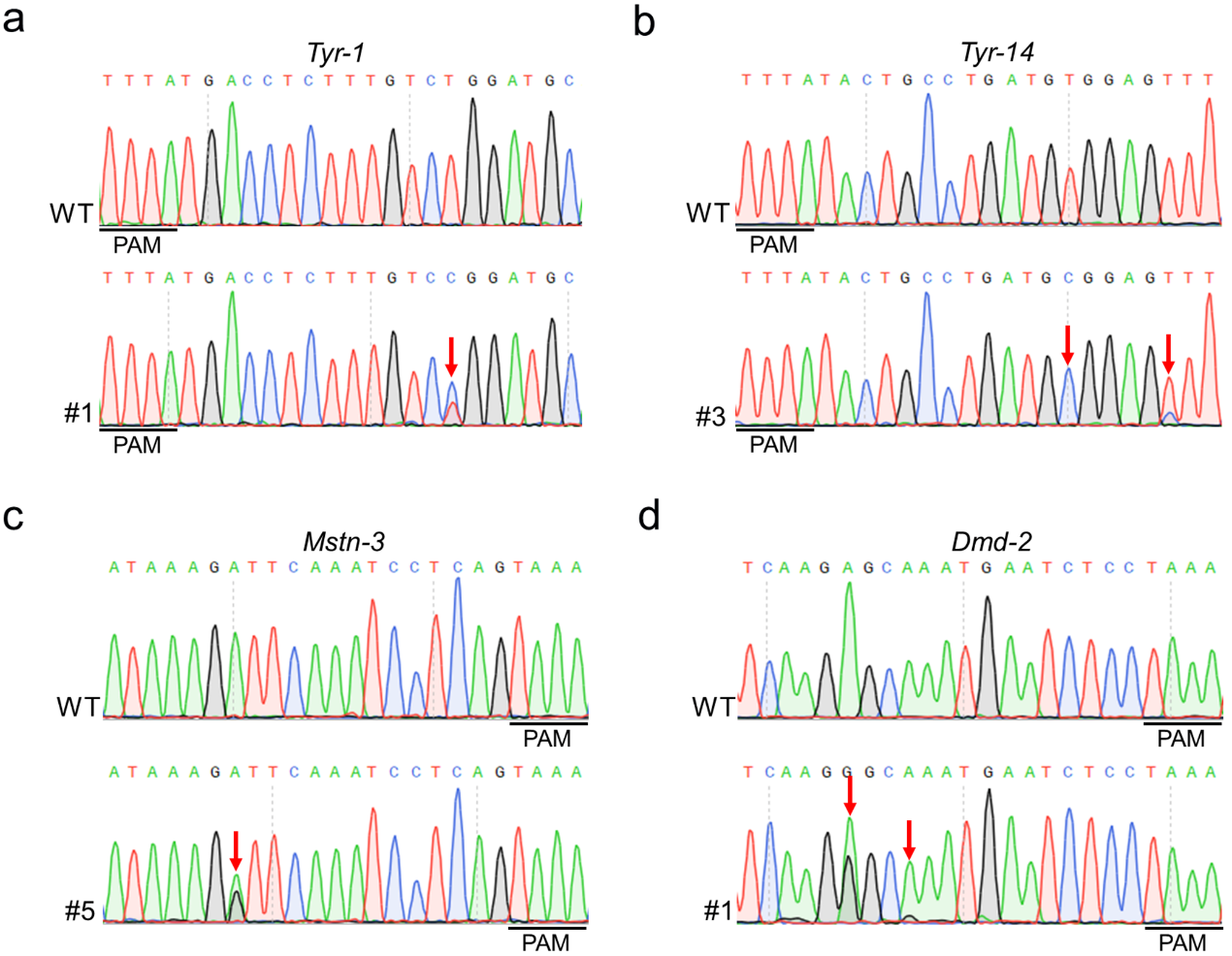


**Figure S4.** Representative sequencing chromatograms of rabbit blastocysts edited at four target sites using the Spy-mac ABEmax system. Targeted A-to-G/T-to-C base editing (red arrows).


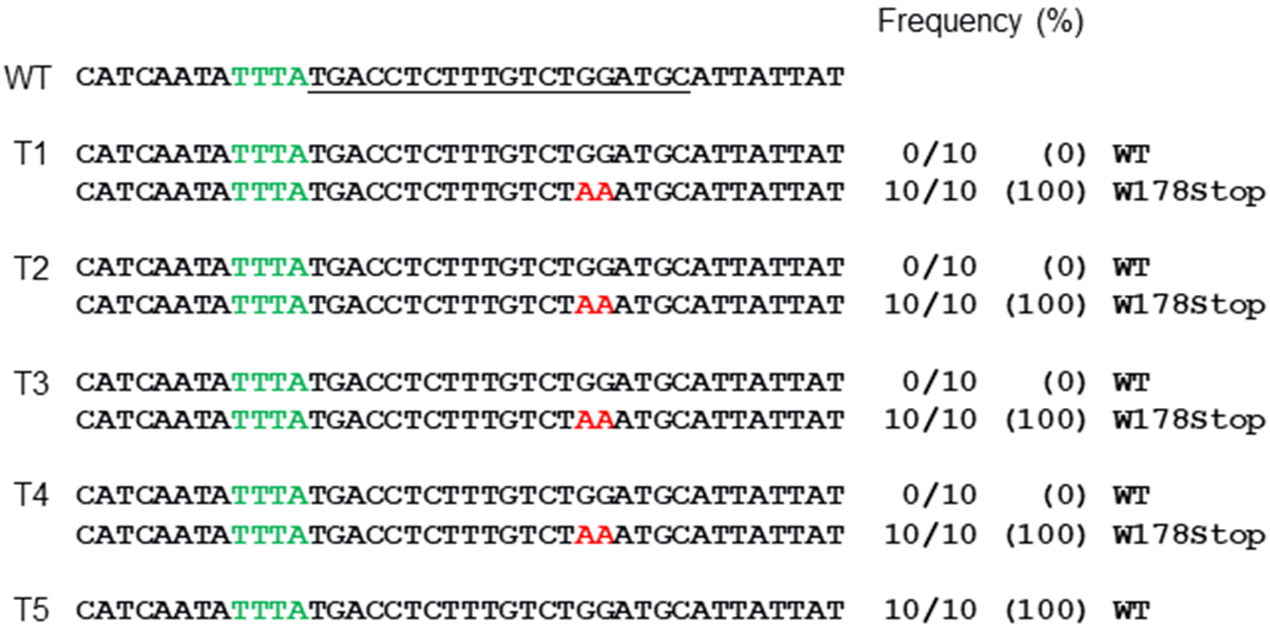


**Figure S5.** Alignment of the mutant sequences of F0 rabbits from T-A cloning at *Tyr*-1. The targeted sequence is underlined. The PAM site and base conversions are shown in green and red, respectively. T1-T5, each F0 rabbit used for T-A cloning. N/N indicates positive colonies out of the total sequenced. The column on the right indicates the frequencies of mutant alleles. WT, wild type.


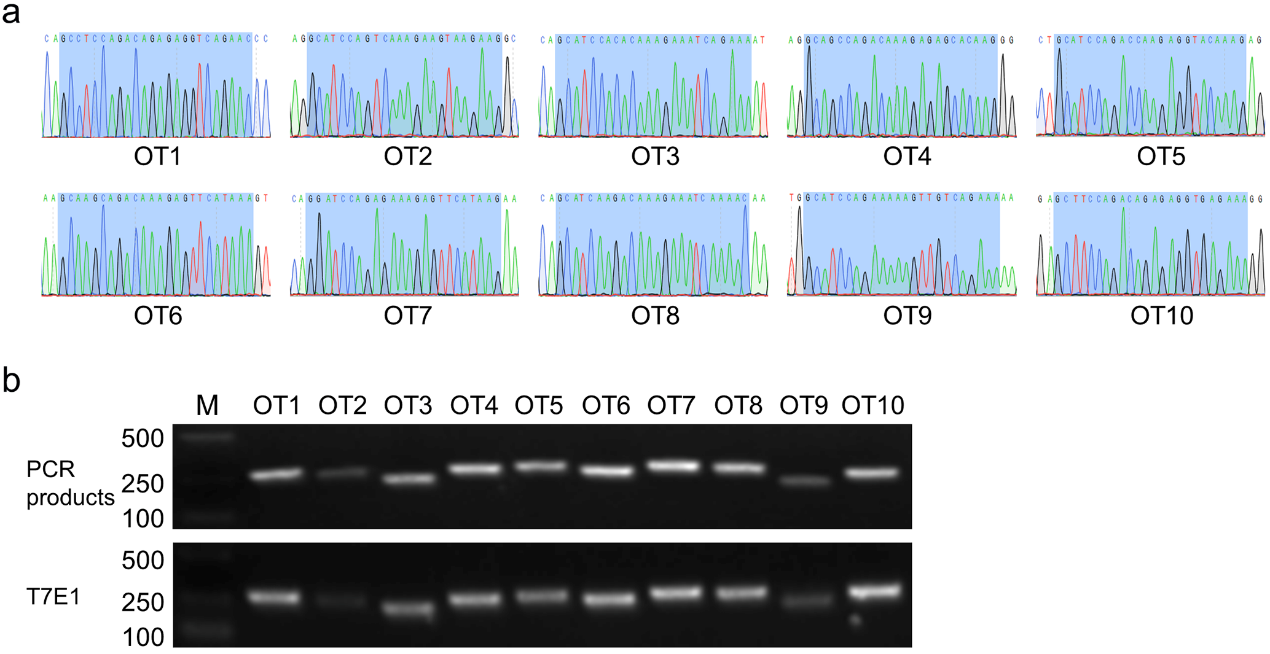


**Figure S6.** Off-target detection in F0 mutant rabbits. **a** Chromatogram sequence analysis of POTs using PCR products for the *Tyr-1* site. A total of 20 bp of the POTs and the PAMs are highlighted. **b** T7E1 cleavage analysis of POTs. M, DL2000.

**Table S1.** Target sites used in this study. Target sequence (black), PAM region (green).

| **PAM** | | **Target site** | **Target site (5’-3’)** |
| --- | --- | --- | --- |
| NAAA | TAAA | *Tyr-1* | TTTATGACCTCTTTGTCTGGATGC |
|  | AAAA | *Tyr-2* | TTTTATACTGCCTGATGTGGAGTT |
|  | GAAA | *Tyr-3* | GAAAGGCGACTCTTGGTGAGGAAA |
|  | CAAA | *Mstn-1* | TTTGAAGCTTTTGGATGGGATTGG |
| NAAT | TAAT | *Mstn-2* | ATTACTGCTCTGGAGAGTGTGAAT |
|  | AAAT | *Tyr-4* | TGAGTGTCTCAGAAAAGGACAAAT |
|  | GAAT | *Tyr-5* | CCAAGAACCTGGTGGAAAAGGAAT |
|  | CAAT | *Tyr-6* | CTAACGCCCCCCTTGGGCCTCAAT |
| NAAG | TAAG | *Dmd-1* | AAAAGCCAGTTAAAAATGTGTAAG |
|  | AAAG | *Tyr-7* | TCCTCCAAGAACCTGGTGGAAAAG |
|  | GAAG | *Tyr-8* | ACACACTGCTTGGGGGATCCGAAG |
|  | CAAG | *Tyr-9* | CCTCGAGCCTGTACCTCCTCCAAG |
| NAAC | TAAC | *Tyr-10* | TGTCAGGACGTCCTTCTGTCTAAC |
|  | AAAC | *Tyr-11* | AGGCGACTCTTGGTGAGGAAAAAC |
|  | GAAC | *Tyr-12* | TCCCCTTCACAGGGGTGGATGAAC |
|  | CAAC | *Tyr-13* | AGAACCTGCCAGTGCTCTGGCAAC |
| TAAA | TAAA | *Tyr-14* | TTTATACTGCCTGATGTGGAGTTT |
|  | TAAA | *Mstn-3* | ATAAAGATTCAAATCCTCAGTAAA |
|  | TAAA | *Dmd-2* | TCAAGAGCAAATGAATCTCCTAAA |

**Table S2.** Generation of *Tyr-1* rabbits using Spy-mac BE4max system.

|  |  |  | **Mutant ratio (%)** | | |
| --- | --- | --- | --- | --- | --- |
| **Target site** | **Embryos**  **transferred** | **No. of offspring** | **No. of mutants** | **No. of**  **homozygous mutants** | **Pups with**  **colour change** |
| *Tyr-1* | 46 | 5 | 4(80) | 4(80) | 4(80) |

**Table S3.** The two oligonucleotide strands used to construct the pUC57-sgRNA vectors.

| **Target site** | **Oligonucleotide 1** | **Oligonucleotide 2** |
| --- | --- | --- |
| *Tyr-1* | TAGGCATCCAGACAAAGAGGTCA | AAACTGACCTCTTTGTCTGGATG |
| *Tyr-2* | TAGGAAACTCCACATCAGGCAGTA | AAACTACTGCCTGATGTGGAGTTT |
| *Tyr-3* | TAGGAAAGGCGACTCTTGGTGAG | AAACCTCACCAAGAGTCGCCTTT |
| *Mstn-1* | TAGGCCAATCCCATCCAAAAGCTT | AAACAAGCTTTTGGATGGGATTGG |
| *Mstn-2* | TAGGATTCACACTCTCCAGAGCAG | AAACCTGCTCTGGAGAGTGTGAAT |
| *Tyr-4* | TAGGTGAGTGTCTCAGAAAAGGAC | AAACGTCCTTTTCTGAGACACTCA |
| *Tyr-5* | TAGGCCAAGAACCTGGTGGAAAAG | AAACCTTTTCCACCAGGTTCTTGG |
| *Tyr-6* | TAGGCTAACGCCCCCCTTGGGCCT | AAACAGGCCCAAGGGGGGCGTTAG |
| *Dmd-1* | TAGGAAAAGCCAGTTAAAAATGTG | AAACCACATTTTTAACTGGCTTTT |
| *Tyr-7* | TAGGTCCTCCAAGAACCTGGTGGA | AAACTCCACCAGGTTCTTGGAGGA |
| *Tyr-8* | TAGGACACACTGCTTGGGGGATCC | AAACGGATCCCCCAAGCAGTGTGT |
| *Tyr-9* | TAGGCCTCGAGCCTGTACCTCCTC | AAACGAGGAGGTACAGGCTCGAGG |
| *Tyr-10* | TAGGTGTCAGGACGTCCTTCTGTC | AAACGACAGAAGGACGTCCTGACA |
| *Tyr-11* | TAGGAGGCGACTCTTGGTGAGGAA | AAACTTCCTCACCAAGAGTCGCCT |
| *Tyr-12* | TAGGTCCCCTTCACAGGGGTGGAT | AAACATCCACCCCTGTGAAGGGGA |
| *Tyr-13* | TAGGAGAACCTGCCAGTGCTCTGG | AAACCCAGAGCACTGGCAGGTTCT |
| *Tyr-14* | TAGGAAACTCCACATCAGGCAGTA | AAACTACTGCCTGATGTGGAGTTT |
| *Mstn-3* | TAGGATAAAGATTCAAATCCTCAG | AAACCTGAGGATTTGAATCTTTAT |
| *Dmd-2* | TAGGTCAAGAGCAAATGAATCTCC | AAACGGAGATTCATTTGCTCTTGA |

**Table S4.** Primers used for genotyping in this study.

| **Target site** | **Primers** | **Sequence (5’-3’)** | **Product size (bp)** |
| --- | --- | --- | --- |
| *Tyr-1*,*Tyr-8* | *Tyr*-F1  *Tyr*-R1 | GCGACTCTTGGTGAGGAAA  AAAGATGCTGGGCTGAGTAG | 459 |
| *Tyr-2~7*,  *Tyr-9~14* | *Tyr*-F2  *Tyr*-R2 | ATCCGCTCAAGCAGGTATTG  GACATAGTCTGGGCTCGTAGTA | 487 |
| *Mstn-1* | *Mstn-1*-F  *Mstn-1*-R | TAGAGGTCAAGGTAACGGACA  GAGACATCTTTGTGGGAGTACAG | 282 |
| *Mstn-2* | *Mstn-2*-F  *Mstn-2*-R | TAGAGGTCAAGGTAACGGACA  GAGACATCTTTGTGGGAGTACAG | 282 |
| *Dmd-1* | *Dmd -1*-F  *Dmd -1*-R | TCTTTCAGCCTGTGACTTCAG  GTGGCTTAGCTAAATCTGTAGGA | 421 |
| *Mstn-3* | *Mstn-3*-F  *Mstn-3*-R | GGCCCAGTGGATCTAAATGAA  AGACTGTCTTTCCTGCTTCTTAC | 449 |
| *Dmd-2* | *Dmd -2*-F  *Dmd -2*-R | TTCTTTCTTTGACAGGGCTACA  ATCCTGACTTAACCACCAATCC | 300 |

**Table S5.** The primers used for identifying potential off-target sites in this study. The mismatched nucleotides are shown in lower case.

| **Potential Off Target Site** | **Number of mismatch** | **Position** | **PCR Primer** |
| --- | --- | --- | --- |
| GCcTCCAGACAgAGAGGTCAGAAC | 2 | chrUn0056:-742533 | OT1-F: ACAAGTCCACGCCTTCAAA  OT1-R: CTGGGAGATCATTCCCGAAATC |
| GCATCCAGtCAAAGAaGTaA  GAAG | 3 | chr1:+78977934 | OT2-F: CTTGGTTCCAACTCAGTCAGTA  OT2-R: TGGCTCTCTAGGTCTCCTTT |
| GCATCCAcACAAAGAaaTCAGAAA | 3 | chr1:+158039264 | OT3-F: CGGCTTTGTGGATCCATTTG  OT3-R: CCCTCTTGGCCTTGATTGT |
| GCAgCCAGACAAAGAGagCACAAG | 3 | chr1:+172972338 | OT4-F: GACATCTGCCTCAGTCTGTTT  OT4-R: GCTTGGGTGTCATTGGTAGT |
| GCATCCAGACcAAGAGGTacAAAG | 3 | chr13:+65576276 | OT5-F: AAAGCTCAGGGACTGGTTAAG  OT5-R: CATCAGAACGGAGAGGAATATAGAG |
| GCAagCAGACAAAGAGtTCATAAA | 3 | chr13:-89356455 | OT6-F: CAGATGGGAGGAAGAATTCTAAGG  OT6-R: AGTGGAGCCTTATGTTGAGATG |
| GgATCCAGAgAAAGAGtTCA  TAAG | 3 | chr14:-129968495 | OT7-F: GGTAACAGCTGGGTGAATCT  OT7-R: GGAGGTTTGGTGGTCCTATTT |
| GCATCaAGACAAAGAaaTCAAAAC | 3 | chr15:-68848622 | OT8-F: CTTCCCATCCGTGGCTATTT  OT8-R: AGGGAAGTCACATCCATCAATC |
| GCATCCAGAaAAAGttGTCA  GAAA | 3 | chr2:+15105964 | OT9-F: CACTCCAGATTGCCCTACTT  OT9-R: TCATGCCAGGTTCTCCTATATTC |
| GCtTCCAGACAgAGAGGTgAGAAA | 3 | chr2:+117299000 | OT10-F: GACAGACATGGAAGGGAGTTT  OT10-R: TCGTATGTGTTCTATGACCTTGG |

**Supplementary sequences**

**Amino acid sequence of Spy-mac BE4max and Spy-mac ABEmax.**

Within the sequences below, NLS sequences are in purple, rAPOBEC1 sequences are in yellow, the Spy Cas9 nickase PIΔ sequence is in gray, the Smac Cas9 PI sequence is in blue, the UGI sequences are in green and the adenine deaminase sequence is in red.

**Spy-mac BE4max amino acid sequence:**

MKRTADGSEFESPKKKRKVSSETGPVAVDPTLRRRIEPHEFEVFFDPRELRKETCLLYEINWGGRHSIWRHTSQNTNKHVEVNFIEKFTTERYFCPNTRCSITWFLSWSPCGECSRAITEFLSRYPHVTLFIYIARLYHHADPRNRQGLRDLISSGVTIQIMTEQESGYCWRNFVNYSPSNEAHWPRYPHLWVRLYVLELYCIILGLPPCLNILRRKQPQLTFFTIALQSCHYQRLPPHILWATGLKSGGSSGGSSGSETPGTSESATPESSGGSSGGSDKKYSIGLAIGTNSVGWAVITDEYKVPSKKFKVLGNTDRHSIKKNLIGALLFDSGETAEATRLKRTARRRYTRRKNRICYLQEIFSNEMAKVDDSFFHRLEESFLVEEDKKHERHPIFGNIVDEVAYHEKYPTIYHLRKKLVDSTDKADLRLIYLALAHMIKFRGHFLIEGDLNPDNSDVDKLFIQLVQTYNQLFEENPINASGVDAKAILSARLSKSRRLENLIAQLPGEKKNGLFGNLIALSLGLTPNFKSNFDLAEDAKLQLSKDTYDDDLDNLLAQIGDQYADLFLAAKNLSDAILLSDILRVNTEITKAPLSASMIKRYDEHHQDLTLLKALVRQQLPEKYKEIFFDQSKNGYAGYIDGGASQEEFYKFIKPILEKMDGTEELLVKLNREDLLRKQRTFDNGSIPHQIHLGELHAILRRQEDFYPFLKDNREKIEKILTFRIPYYVGPLARGNSRFAWMTRKSEETITPWNFEEVVDKGASAQSFIERMTNFDKNLPNEKVLPKHSLLYEYFTVYNELTKVKYVTEGMRKPAFLSGEQKKAIVDLLFKTNRKVTVKQLKEDYFKKIECFDSVEISGVEDRFNASLGTYHDLLKIIKDKDFLDNEENEDILEDIVLTLTLFEDREMIEERLKTYAHLFDDKVMKQLKRRRYTGWGRLSRKLINGIRDKQSGKTILDFLKSDGFANRNFMQLIHDDSLTFKEDIQKAQVSGQGDSLHEHIANLAGSPAIKKGILQTVKVVDELVKVMGRHKPENIVIEMARENQTTQKGQKNSRERMKRIEEGIKELGSQILKEHPVENTQLQNEKLYLYYLQNGRDMYVDQELDINRLSDYDVDHIVPQSFLKDDSIDNKVLTRSDKNRGKSDNVPSEEVVKKMKNYWRQLLNAKLITQRKFDNLTKAERGGLSELDKAGFIKRQLVETRQITKHVAQILDSRMNTKYDENDKLIREVKVITLKSKLVSDFRKDFQFYKVREINNYHHAHDAYLNAVVGTALIKKYPKLESEFVYGDYKVYDVRKMIAKSEQEIGKATAKYFFYSNIMNFFKTEITLANGEIRKRPLIETNGETGEIVWDKGRDFATVRKVLSMPQVNIVKKTEIQTVGQNGGLFDDNPKSPLEVTPSKLVPLKKELNPKKYGGYQKPTTAYPVLLITDTKQLIPISVMNKKQFEQNPVKFLRDRGYQQVGKNDFIKLPKYTLVDIGDGIKRLWASSKEIHKGNQLVVSKKSQILLYHAHHLDSDLSNDYLQNHNQQFDVLFNEIISFSKKCKLGKEHIQKIENVYSNKKNSASIEELAESFIKLLGFTQLGATSPFNFLGVKLNQKQYKGKKDYILPCTEGTLIRQSITGLYETRVDLSKIGEDSGGSGGSGGSTNLSDIIEKETGKQLVIQESILMLPEEVEEVIGNKPESDILVHTAYDESTDENVMLLTSDAPEYKPWALVIQDSNGENKIKMLSGGSGGSGGSTNLSDIIEKETGKQLVIQESILMLPEEVEEVIGNKPESDILVHTAYDESTDENVMLLTSDAPEYKPWALVIQDSNGENKIKMLSGGSKRTADGSEFEPKKKRKV

**Spy-mac ABEmax amino acid sequence:**

MKRTADGSEFESPKKKRKVSEVEFSHEYWMRHALTLAKRAWDEREVPVGAVLVHNNRVIGEGWNRPIGRHDPTAHAEIMALRQGGLVMQNYRLIDATLYVTLEPCVMCAGAMIHSRIGRVVFGARDAKTGAAGSLMDVLHHPGMNHRVEITEGILADECAALLSDFFRMRRQEIKAQKKAQSSTDSGGSSGGSSGSETPGTSESATPESSGGSSGGSSEVEFSHEYWMRHALTLAKRARDEREVPVGAVLVLNNRVIGEGWNRAIGLHDPTAHAEIMALRQGGLVMQNYRLIDATLYVTFEPCVMCAGAMIHSRIGRVVFGVRNAKTGAAGSLMDVLHYPGMNHRVEITEGILADECAALLCYFFRMPRQVFNAQKKAQSSTDSGGSSGGSSGSETPGTSESATPESSGGSSGGSDKKYSIGLAIGTNSVGWAVITDEYKVPSKKFKVLGNTDRHSIKKNLIGALLFDSGETAEATRLKRTARRRYTRRKNRICYLQEIFSNEMAKVDDSFFHRLEESFLVEEDKKHERHPIFGNIVDEVAYHEKYPTIYHLRKKLVDSTDKADLRLIYLALAHMIKFRGHFLIEGDLNPDNSDVDKLFIQLVQTYNQLFEENPINASGVDAKAILSARLSKSRRLENLIAQLPGEKKNGLFGNLIALSLGLTPNFKSNFDLAEDAKLQLSKDTYDDDLDNLLAQIGDQYADLFLAAKNLSDAILLSDILRVNTEITKAPLSASMIKRYDEHHQDLTLLKALVRQQLPEKYKEIFFDQSKNGYAGYIDGGASQEEFYKFIKPILEKMDGTEELLVKLNREDLLRKQRTFDNGSIPHQIHLGELHAILRRQEDFYPFLKDNREKIEKILTFRIPYYVGPLARGNSRFAWMTRKSEETITPWNFEEVVDKGASAQSFIERMTNFDKNLPNEKVLPKHSLLYEYFTVYNELTKVKYVTEGMRKPAFLSGEQKKAIVDLLFKTNRKVTVKQLKEDYFKKIECFDSVEISGVEDRFNASLGTYHDLLKIIKDKDFLDNEENEDILEDIVLTLTLFEDREMIEERLKTYAHLFDDKVMKQLKRRRYTGWGRLSRKLINGIRDKQSGKTILDFLKSDGFANRNFMQLIHDDSLTFKEDIQKAQVSGQGDSLHEHIANLAGSPAIKKGILQTVKVVDELVKVMGRHKPENIVIEMARENQTTQKGQKNSRERMKRIEEGIKELGSQILKEHPVENTQLQNEKLYLYYLQNGRDMYVDQELDINRLSDYDVDHIVPQSFLKDDSIDNKVLTRSDKNRGKSDNVPSEEVVKKMKNYWRQLLNAKLITQRKFDNLTKAERGGLSELDKAGFIKRQLVETRQITKHVAQILDSRMNTKYDENDKLIREVKVITLKSKLVSDFRKDFQFYKVREINNYHHAHDAYLNAVVGTALIKKYPKLESEFVYGDYKVYDVRKMIAKSEQEIGKATAKYFFYSNIMNFFKTEITLANGEIRKRPLIETNGETGEIVWDKGRDFATVRKVLSMPQVNIVKKTEIQTVGQNGGLFDDNPKSPLEVTPSKLVPLKKELNPKKYGGYQKPTTAYPVLLITDTKQLIPISVMNKKQFEQNPVKFLRDRGYQQVGKNDFIKLPKYTLVDIGDGIKRLWASSKEIHKGNQLVVSKKSQILLYHAHHLDSDLSNDYLQNHNQQFDVLFNEIISFSKKCKLGKEHIQKIENVYSNKKNSASIEELAESFIKLLGFTQLGATSPFNFLGVKLNQKQYKGKKDYILPCTEGTLIRQSITGLYETRVDLSKIGEDSGGSKRTADGSEFEPKKKRKV
